# A Randomized, Double-Blind Trial of Lisinopril and Losartan for the Treatment of Cardiomyopathy in Duchenne Muscular Dystrophy

**DOI:** 10.1371/currents.md.2cc69a1dae4be7dfe2bcb420024ea865

**Published:** 2013-12-12

**Authors:** Hugh D. Allen, Kevin M Flanigan, Philip T. Thrush, Igor Dvorchik, Han Yin, Charles Canter, Anne M. Connolly, Mark Parrish, Craig M. McDonald, Elizabeth Braunlin, Steven D. Colan, John Day, Basil Darras, Jerry R. Mendell

**Affiliations:** Center for Gene Therapy, Heart Center, and Wellstone Cooperative Muscular Dystrophy Research Center, Nationwide Children’s Hospital, Columbus, Ohio, USA; Department of Pediatrics, The Ohio State University, Columbus, Ohio, USA; Texas Children’s Hospital, Baylor College of Medicine, Houston, Texas, USA; Departments of Neurology and Pediatrics, The Ohio State University, Columbus, Ohio, United States; Center for Gene Therapy, Nationwide Children's Hospital, Columbus, Ohio, United States; Center for Gene Therapy, Heart Centre, and Wellstone Cooperative Muscular Dystrophy Research Center, Nationwide Children's Hospital, Columbus, Ohio, USA; Department of Pediatrics, The Ohio State University, Columbus, Ohio, USA; Center for Mathematical Medicine, Nationwide Children’s Hospital, Columbus, Ohio, USA; Department of Pediatrics, The Ohio State University, Columbus, Ohio, USA; Center for Mathematical Medicine, Nationwide Children’s Hospital, Columbus, Ohio, USA; Department of Pediatrics, The Ohio State University, Columbus, Ohio, USA; St. Louis Children’s Hospital, Washington University College of Medicine, St. Louis, Missouri, USA; St. Louis Children’s Hospital, Washington University College of Medicine, St. Louis, Missouri, USA; University of California Davis, Davis, California, USA; University of California Davis, Davis, California, USA; The University of Minnesota College of Medicine, Minneapolis, Minnesota, USA; Boston Children’s Hospital, Harvard Medical School, Boston, USA; The University of Minnesota College of Medicine, Minneapolis, Minnesota, USA; Boston Children’s Hospital, Harvard College of Medicine, Boston, Massachusetts, USA; Departments of Neurology and Pediatrics, The Ohio State University, Columbus, Ohio, USA; Center for Gene Therapy, Nationwide Children's Hospital, Columbus, Ohio, USA

## Abstract

Objectives: This study sought to compare the effectiveness and safety of an angiotensin converting enzyme inhibitor (ACE-I) (lisinopril) vs. an angiotensin receptor blocker (ARB) (losartan) for the treatment of cardiomyopathy (CM) in boys with Duchenne muscular dystrophy (DMD). Background: Development of CM is universal in boys with DMD. ACE-I and ARB have both been suggested as effective treatment options. ARBs have been associated with skeletal muscle regeneration in a mouse model of DMD. The question of which, if either, is more effective for CM treatment in DMD remains. The purpose of this multicenter double-blind prospective study was to compare efficacy and safety of lisinopril versus losartan in the treatment of newly diagnosed CM in boys with DMD. Methods: Echocardiographic technician inter- and intraobserver variability were tested on 2 separate days on 2 different boys with DMD CM. Results were compared with paired t-testing. Twenty-two boys with newly diagnosed DMD CM (echocardiographic ejection fraction (EF) 10% EF drop. Three boys in the aCE-I group had 3 visits, due to study funding termination. Two were withdrawn because of low EF. All their data are included in the analysis for as long as they remained in the study. Mean EF’s were similar at baseline (47.5%- ACE-I, 48.4%- ARB). After 1 year each group significantly improved to 54.6% and 55.2% respectively (p=.02). There was no difference between the 2 treatment groups at 1 year. Conclusions: Inter-observer and intra-observer reliability studies showed no differences between echocardiographers on serial examinations. EF improved equally in the two groups. There is no therapeutic difference in EF improvement between lisinopril and losartan over the one-year duration for treatment of boys with DMD-related CM. Trial Registration: ClinicalTrials.gov NCT01982695

## Introduction

Cardiomyopathy (CM) is a universal sequela of Duchenne muscular dystrophy (DMD), with onset at a mean age of 14-15 years. 1 ^-^ 6 With improved pulmonary care, DMD patients now live longer 7 only to succumb from the complications of CM, including heart failure and dysrhythmias. As these patients are usually wheelchair-dependent and fairly immobile by this time, their cardiomyopathy rarely causes symptoms. Diagnosis is generally made by echocardiography; with most agreeing that an ejection fraction (EF) < 55% is consistent with the diagnosis of CM.

Treatment options vary. Digoxin has mostly dropped from favor, replaced by most cardiologists with either an angiotensin converting enzyme inhibitor (ACE-I) (commonly lisinopril) 1 ^-^ 3 ^,^ 8 ^-^ 11 or angiotensin II receptor blocker (ARB) (commonly losartan) 12 ^-^ 16. Although we have recently shown that ACE-I treatment improves the cardiomyopathy for a time, adjunctive β-blocker treatment does not seem to have a significant effect on its improvement, however this study was not designed or powered to test the effect of beta blocker alone 2.

Losartan has been demonstrated to improve congestive heart failure symptoms and ejection fraction in adults with ischemic heart disease 12 ^,^ 13 ^,^ 16. Animal studies have demonstrated that ARB improves cardiac function in dystrophic mice 17 ^-^ 19. They have also been reported to enhance skeletal muscle regeneration, but this has not been proven in humans 17. Nevertheless, because of the skeletal muscle results seen in mice, some cardiologists prefer using ARB to treat DMD CM.

β-blocker therapy is often employed as an adjunct to treat the heart failure or to reduce average heart rates due to the accompanying disordered automaticity 20 ^-^ 23.

We hypothesized that lisinopril and losartan have equal efficacy in the treatment of DMD CM. The specific aims were to compare the effectiveness of ACE-I vs. ARB treatment in a double-blinded prospective study of DMD boys who had new onset cardiomyopathy defined by an EF of less than 55% by echocardiography.

Another aim was to validate echocardiographer data acquisition and measurement to assure accuracy of the echocardiographic information.

## Methods

Echo standardization and validation

Echocardiographic technicians from each of the 5 participating centers traveled to the primary site for reliability testing, during which each examined the same two DMD boys on two separate days. The two boys, aged 9 years and 17 years, were asked to volunteer for these studies after parents signed IRB approved consent and the patients signed assent. The boys chosen for this testing were known to be difficult to examine because of body habitus. Each boy was compensated monetarily ($50) for his participation. A cardiologist made patient assignments and monitored these sessions (HDA). A standard protocol was followed with the technicians measuring left ventricular dimensions and planimeterizing the 4 chamber apex view for derivation of EF using Simpson’s rule. The examinations were blinded and were then re-read and remeasured by one of the investigators (HDA) and data were recorded.

Inter- and intra-examiner results were compared using paired t-testing.

Study entry

DMD patients of any age were considered for enrollment. Inclusion criteria included a clinical course consistent with DMD, a proven mutation of the DMD gene or muscle dystrophin levels of <5% on muscle biopsy, a Doppler echocardiogram with EF <55% within 30 days of study initiation, and the ability to cooperate with testing.

Glucocorticoid treatment was acceptable, including either daily or weekend dosing of prednisone or deflazacort. Treatment with metoprolol or an equivalent beta-blocker was also acceptable. It was previously established that concomitant beta-blocker therapy did not seem to alter EF improvement results in patients treated with an ACE-I , although the study cited was not designed or powered to test the effect of beta blocker alone 2. We did not have genotypic information from all centers. We did not have information regarding ambulatory status or respiratory support.

Patients who were currently taking either lisinopril ≤ 5 mg, losartan ≤ 25 mg or enalapril ≤ 5 mg were eligible for enrollment after a two-week washout if their repeat echocardiogram showed an EF > 40% but < 55% (considered as a washout cohort). Exclusion criteria included patients with an EF ≥ 55% or ≤ 40% after washout; current treatment with > 5 mg lisinopril, > 25 mg losartan, or > 5 mg enalapril; or manifest skeletal deformities or pulmonary anatomical variants that precluded consistent measures by Doppler echocardiography.

All subjects were enrolled under Institutional Review Board-approved protocols. Consent (adults) and assent (boys between ages 9-18) forms were signed by all participants, and stored at Nationwide Children’s Hospital (NCH) as well as at participating sites.The study's IRB number is IRB09-00051.

Echo data

All echoes were initally read at the patient’s home site by the participating pediatric cardiologist. They were then cross-read and confirmed at the primary site by 2 of the authors (HDA, PTT). EF was measured using the biplane Simpson’s rule using images obtained from the apical 4 chamber view 24. The bullet method (area-length) was used for EF calculation in 2 patients from one site 25. The echo core site readers and the home site physicians and coordinators had absolutely no knowledge as to which drug the patient was receiving.

All echo-derived data were recorded on a standardized sheet, and data were finalized and entered into a dedicated database at the primary site (NCH Bioinformatics Core). All data were stored in strict adherence to HIPAA regulations regarding patient privacy. Numbers were provided to individual centers and patients were enrolled using an appropriate code.

Drug protocol

Randomization was performed by the NCH Investigational Drug Pharmacy. With entry into the study, the boys were given a capsule that contained either lisinopril or losartan, prepared by the research pharmacist at Nationwide Children’s Hospital. The contents of the capsule looked identical, no matter which drug they contained. None of the study investigators (the cardiologist, neurologist, and site study coordinators) had knowledge of which drug was in the capsule.

Lisinopril dosage was as close to 0.07 mg/kg as possible; losartan dosage was as close to 0.7 mg/kg as possible; generally at 5 mg/day (lisinopril) or 25 mg/day (losartan). The capsules were taken daily and the subjects returned for follow up at 4 monthly intervals. Any siblings enrolled in the study received the same drug (one occurrence).

Examination and history were standardized, and most subjects had at least four evaluations (baseline, and three subsequent visits 4 months apart). At each follow-up visit, a standard questionnaire included inquiry into the most common potential adverse effects, including dizziness, cough, syncope, and headache. Blood pressures were measured and the patient was examined. The container containing the capsules was returned and any remaining capsules were returned to the research pharmacist. The patient was then provided with a 4-month refill of capsules, with dosage adjusted based on the result of the repeat echocardiogram. No change in dosage was made if the EF was within 5% of the original EF, dosage was doubled if the EF decreased 5-10%, and the patient was discontinued from the study if the EF decreased > 10%. Allergy to the medication was another reason for termination.

Treating physicians were unblinded only at the termination of the study, or if the patient was discharged from the study, in which case the treating cardiologist provided ongoing care was informed as to which drug was employed. The investigators at the primary site remained blinded to the drug used.

Data analysis

Ejection fraction was the primary data endpoint. All the outcome measures were normally distributed. EF was serially compared at each visit with baseline using paired t- tests within each drug group. Baseline EF and at the last (usually 4^th)^ visit were compared between the ACE-I and ARB groups using two sample t-test and corroborated with mixed model analysis. Baseline age was nonparametric and was compared using Wilcoxon rank sum test. Comparison of the presence of steroid treatment or β-blocker treatment was made using Fisher’s exact test. The relative effect of two medications on EF was analyzed by mixed model adjusting on age, steroid status, visit number and baseline EF.

**Enrollment, Allocation, Follow-up, and Analysis of Trial Participants:**
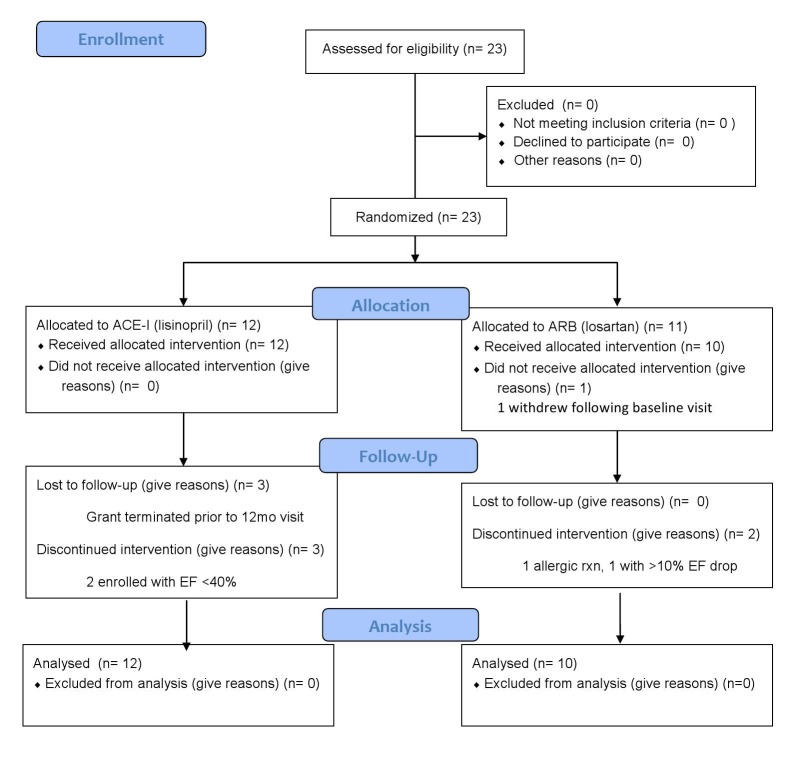

## Results

Population

Twenty-three boys were enrolled into the study. One (losartan assignment) immediately withdrew and of the remaining 22 patients, 12 were randomized to the lisinopril group and 10 to the losartan group. Two boys, both in the losartan group, were removed from the study, 1 because of an allergic reaction (hives) and 1 who exceeded the safety standard of a >10% EF decrease. Five boys in the lisinopril group did not complete one year; three had their 3^rd^ visit, then the study was terminated due to funding cessation. Two were enrolled with EF below 40% at one of the sites and were terminated at their second visit when this deviation from the protocol was discovered. All data are included in the results and analysis until the time of removal or cessation.

Age distribution between the 2 groups was similar, with a median of 12.5 years (range, 10 to 21 y) for the lisinopril group and a median of 15.5 years (range, 7 to 27 y) for the losartan group.

Eight in the lisinopril group and 2 in the losartan group were taking steroids (p=.04), and 0 in the lisinopril group and 2 in the losartan group were treated with β-blockers (p=NS).

Inter- and intra-observer reliability studies

There was no difference in the mean echo measurement values derived from the same patient on 2 different days by the same technician (p=ns). There was no significant difference in values on the same patient among the technicians on 2 separate days (p=ns). (Table I).

No statistically significant differences were noted.

**Table I. Comparison of inter- and intra-observer variability in measurement of EF (%).** No statistically significant differences were noted.

Technician	Patient 01Day 1	Day 2	Patient 02Day 1	Day 2
1	48.0	49.0	62.0	67.0
2	48.1	47.1	67.3	64.1
3	46.0	50.2	62.7	62.7
4	50.0	50.0	63.0	67.0
5	48.0	50.0	63.0	67.0
MEAN	48.0	49.3	64.6	65.5
SD	1.4	1.3	2.8	2.0

Ejection Fraction

Mean EFs were similar at baseline (47.5% for the lisinopril group and 47.7% for the losartan group; p=0.93) (Table II). After one year, there was a significant improvement in EF from baseline with use of either medication, to 54.6% in the lisinopril group (p=0.02) and to 55.2% in the losartan group (p=.03). However, there was no difference between the 2 treatment groups at one year (p=0.86) (Table III) (Figure 2).

**Serial data for both groups. Lisinopril in red, losartan in blue.**
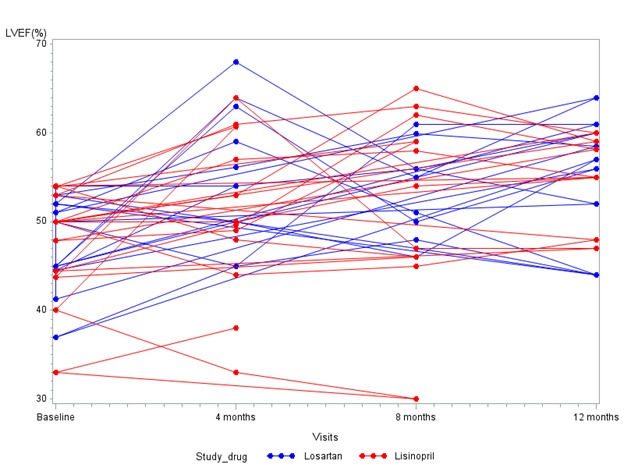
EF is shown on the ordinate and time on the abscissa.

**Table II. Baseline Data:** LVEF = left ventricular ejection fraction; SD = standard deviation; NS = not significant, Rx = treatment. No difference was found between two treatments (p=0.29) using a mixed model adjusting for age, steroid status, visit number and baseline EF.

	Lisinopril group (n=12)	Losartan group (n=11)	p-value
Mean LVEF, % (SD)	47.5 (6.25)	47.7(5.49)	0.93 (NS)
Age, years Q1-Q3Range	12.5(11 – 18)(10 - 21)	15.5(14 – 18)(7 - 27)	0.52 (NS)
Steroid Rx, n	8	2	0.04
β-Blocker, n	0	1	0.48 (NS)

**Table IIIA. Ejection fraction (%) at each visit.** Bold and italcized values were obtained from patients on β-blocker at the time of the visit. * = subject was not on corticosteroid therapy at the baseline visit but was on steroids for each visit thereafter.

		Ejection Fraction (%) at visit			
Patient ID	Steroid	baseline	4 months	8 months	12 months
1	N	52	50	46	57
2	N	37	45	48	44
4	N	52	68	56	52
6	N	50	***45***	***61***	***61***
9	Y	45	64	55	64
12	Y	51	59	51	44
13	N	53	56	60	59
14	N	41			
16	N	54	54	56	60
17	*	45	63	50	56
21	N	***45***	***54***		

3	N	50	44	45	***48***
5	Y	53	***61***	***63***	***60***
7	Y	50	***53***	***65***	***59***
8	Y	50	50	54	55
10	Y	50	***57***	***58***	***55***
11	N	54	***48***	***46***	
15	Y	44	64	47	47
18	Y	44	50	62	58
19	Y	48	49	59	
20	Y	54	61		
22	N	40	33	30	
23	N	33	38		

**Table IIIB. Serial mean ejection fraction values - Grouped data:** LVEF = left ventricular ejection fraction

	Lisinopril		Losartan	
Visit	N	mean LVEF (%)	N	mean LVEF (%)
**Baseline**	12	47.5	10	48.3
**4 months**	12	50.6	10	55.8
**8 months**	10	52.9	9	53.7
**12 months**	7	54.6	9	55.2

## Discussion

The main finding of this prospective double-blinded study is that there is no therapeutic difference between lisinopril and losartan over one-year’s duration of treatment of boys with DMD-related CM. Each drug significantly improved systolic function at one year. Our results suggest that since the drugs are equally effective either can be safely used to treat the CM of DMD.One of the losartan patients was removed from the study due to an allergic reaction. His data are included. The other patient dropped below the acceptable EF decrease for continued blinded administration, but his was the only discontinuance of 10 total patients receiving losartan. We feel that this situation is more consistent with the natural history of the disease than drug toxicity; nonetheless, his data are included in the analysis until the time that he was removed from the investigation.Three of the lisinopril treated boys had three visits but did not have a fourth due to termination of funding of the study. Two lisinopril treated boys from the same center did not fit the protocol as their initial EF was less than 40% and were discontinued when this was discovered.

Our results also confirm the utility of echocardiography in DMD trials. In the current era of advanced imaging, such as cardiac MRI, it can be tempting to dismiss echocardiography as an imaging modality in this patient population due to the difficulty of obtaining adequate imaging. We do not intend to underscore the importance of advanced imaging in this population, as it can yield additional information such as ventricular myocardial fibrosis and strain/strain rate analysis. However, cardiac MRI imaging does have drawbacks, especially in the research arena. A cardiac MRI, is typically a longer study, and as such may require sedation/anesthesia in order to obtain optimal imaging. In addition, if gadolinium is given to evaluate for late-gadolinium enhancement, a peripheral IV is required. Also, there is additional cost required in obtaining a cardiac MRI.

Our inter-rater and intra-rater reliability studies showed no differences between echocardiographers on serial examinations. No study was cancelled due to inability to perform the examination, even on patients who were difficult to position, demonstrating that echocardiography can be utilized to monitor CM in this challenging patient population. While echocardiography may not be suitable to all cardiac research endeavors in this population, our results demonstrate that reliable and reproducible echocardiographic data can be obtained, and echocardiography should not be dismissed as an imaging modality, even in the patient with DMD who has difficult acoustic windows.

Our study did have several limitations. First, this study only evaluates the short-term benefit of either drug over the course of 1 year. Many patients with DMD are treated for CM for far longer than 1 year, but given our results, we are unable to speculate regarding the long-term benefits of either medication. We would expect that given the progressive nature of the disease, CM would continue to progress in both groups, and that one of these medications may prove to delay progression better than the other.

Additionally, while the study is randomized and double-blinded, our numbers are still small despite this being a multi-center study. This is likely limited, in part, by the inclusion criteria of a required washout period for those on lower-dose lisinopril or losartan and by the exclusion of those patients on higher doses of either drug.

Given our entry criteria to evaluate the effects of these drugs on boys with CM associated with DMD, we are also unable to predict whether one of these medications may be cardioprotective if initiated prior to the development of gross cardiac dysfunction.

In conclusion, there is no difference between the effects of lisinopril versus losartan on EF improvement in DMD boys with CM. The drugs were safe. Thus, this study opens the opportunity to study muscle function in younger boys with DMD to see if the skeletal muscle improvement seen in the mdx mouse following treatment with an ARB might also be demonstrated in DMD boys. 17

## Competing Interests

The authors have declared that no competing interests exist.

## Correspondence

Hugh D. Allen MD, Heart Center, Texas Children’s Hospital, 6621 Fannin St. Suite 19469, Houston, TX, 77030

E-mail: hdallen@texaschildrens.org

## Author Contributions

Drs. Allen and Mendell conceived the study, wrote the grant oversaw data collection, and wrote the paper. Dr. Allen saw all Nationwide Children’s patients, re-read all echoes and coordinated the study.

Dr. Flanigan reviewed the data, reviewed the paper and coordinated statistical testing

Dr. Thrush reviewed all echoes, evaluated patients, reviewed the paper and helped with its development.

Drs. Dvorchik and Yin did the statistical analysis and participated in writing the paper.

Drs. Canter and Connolly were site evaluators at St. Louis Children’s Hospital, provided patients, performed and read echoes and reviewed the paper.

Drs. Parrish and Mcdonald were site evaluators at UC Davis, provided patients, performed and read echoes and reviewed the paper.

Drs. Braunlin and Day were site evaluators at the University of Minnesota, provided patients, performed and read echoes and reviewed the paper.

Drs. Colan and Darras were site evaluators at Boston Children’s Hospital, provided patients, performed and read echoes and reviewed the paper.

## Appendix 1

### Consort Checklist

Download PDF

### Trial Protocol

Download PDF

## References

[1] Allen HD, Mendell JR, and Hoffman TM. The Heart in Muscular Dystrophies; Chapter 60 in Moss and Adams’ Heart Disease in Infants, Children, and Adolescents, Including the Fetus and Young Adult, 7th Ed. Phildadelphia, Wolters Kluwer/Lippincott Williams and Wilkins, 2008.

[2] Viollet, Laurence PhD, Thrush, Philip T. MD, Flanigan ,Kevin M. MD, Mendell, Jerry R. MD, Allen, Hugh D MD. “Effects of Angiotensin-Converting Enzyme Inhibitors and/or Beta Blockers on the Cardiomyopathy in Duchenne Muscular Dystrophy”. Am J Cardiol 2012; 110(1):98-102. Epub ahead of print, 3/29/12. 10.1016/j.amjcard.2012.02.064 22463839

[3] Allen HD, Thrush PT, Hoffman TM, Flanigan KM, Mendell JT: Cardiac Management in Neuromuscular Disease. 2012; Phys Med Rehabil Clin N Am 23:855-68. 10.1016/j.pmr.2012.08.001 23137741

[4] Duboc D, Meune C, Lerebours G, Devaux JY, Vaksmann G, Becane HM. Effect of perindopril on the onset and progression of left ventricular dysfunction in Duchenne muscular dystrophy. J Am Coll Cardiol 2005; 45: 855-857. 10.1016/j.jacc.2004.09.078 15766818

[5] Hor KN, Wanasapura J, Markham LW, et al. Circumferential Strain Analysis Identifies Strata of Cardiomyopathy in Duchenne Muscular Dystrophy. A Cardiac Magnetic Resnonance Tagging Study. J Am Coll Cardiol 2009; 53: 1204-10. 10.1016/j.jacc.2008.12.032 PMC2709409 19341862

[6] Puchalski MD, Williams RV, Askovich B, et al. Late gadolinium enhancement: precursor to cardiomyopathy in Duchenne muscular dystrophy. Int J Cardiovasc Imaging 2009; 25: 57-63. 10.1007/s10554-008-9352-y PMC2746925 18686011

[7] Eagle M., Bourke J, Bullock R, Gibson M, Mehta J, Giddings D, Straub V, Bushby K. Managing Duchenne muscular dystrophy-the additive effect of spinal surgery and home nocturnal ventilation in improving survival. Neuromuscul Disord 2007: 17 (6):470-5. Epub 2007 May 8. 10.1016/j.nmd.2007.03.002 17490881

[8] Bosser G, Lucron H, Lethor JP, et al. Evidence of early impairments in both right and left ventricular inotropic reserves in children with Duchenne’s muscular dystrophy. Am J Cardiol 2004; 93:724–727. 10.1016/j.amjcard.2003.12.005 15019877

[9] Jefferies JL, Eidem BW, Belmont JW, et al. Genetic predictors and remodeling of dilated cardiomyopathy in muscular dystrophy. Circulation 2005; 112:2799–2804. 10.1161/CIRCULATIONAHA.104.528281 16246949

[10] Naruse H, Miyagi J, Arii T, et al. The relationship between clinical stage, prognosis and myocardial damage in patients with Duchenne-type muscular dystrophy: Five-year follow-up study. Ann Nucl Med 2004; 18: 203–208. 10.1007/BF02985001 15233281

[11] Stöllberger C, Finsterer J. Can perindopril delay the onset of heart failure in Duchenne muscular dystrophy? J Am Coll Cardiol 2005; 46:1781. 10.1016/j.jacc.2005.08.004 16256886

[12] Guazzi, M, et al Comparison in changes of respiratory function and exercise oxygen uptake with losartan versus enalapril in congestive heart failure due to ischemic or dilated cardiomyopathy. Am J Cardiol 1997; 80:1572-76. 10.1016/s0002-9149(97)00781-9 9416938

[13] Gurlek A, et al. Effect of losartan on circulating TNFalpha levels and left ventricular systolic performance in patients with heart failure. J Cardiovasc Risk 2001; 8: 279 10.1177/174182670100800506 11702033

[14] Yamazaki T, et al. A new therapeutic strategy for hypertrophic nonobstructive cardiomyopathy in humans. A randomized and prospective study with an angiotensin II receptor blocker. Int Heart J 2007; 48:715-24. 10.1536/ihj.48.715 18160763

[15] Bahk T, et al. Comparison of angiotensin converting enzyme inhibition and angiotensin II receptor blockade for prevention of experimental autoimmune myocarditis. Int J Cardiol 2008; 125:85-93. 10.1016/j.ijcard.2007.04.062 PMC2488158 17588693

[16] Ozdemir M, et al. Losartan Improves Heart Rate Variability and Heart Rate Turbulence in Heart Failure Due to Ischemic Cardiomyopathy. J Cardiac Fail 2007; 13:812-817. 10.1016/j.cardfail.2007.08.002 18068613

[17] Cohn RD, van Erp C, Habashi JP, Soleimani AA, Klein EC, Lisi MT et al. Angiotensin II type 1 receptor blockade attenuates TGF-beta-induced failure of muscle regeneration in multiple myopathic states. Nat Med 2007;13: 204-210. 10.1038/nm1536 PMC3138130 17237794

[18] Spurney CF, Sali A, Guerron AD, et al. Losartan decreases cardiac muscle fibrosis and improves cardiac function in dystrophin-deficient mdx mice. J Cardiovasc Pharmacol Ther 2011; 16:87-95. 10.1177/1074248410381757 PMC4147941 21304057

[19] Bish LT, Yarchoan M, Sleeper MM, et al. Chronic losartan administration reduces mortality and preserves cardiac but not skeletal muscle function in dystrophic mice. PLoS One 2011; 6:e20856. 10.1371/journal.pone.0020856 PMC3120761 21731628

[20] Lanza GA, Dello Russo A, Giglio V, et al. Impairment of cardiac autonomic function in patients with Duchenne muscular dystrophy: Relationship to myocardial and respiratory function. Am Heart J 2001; 141:808–812. 10.1067/mhj.2001.114804 11320370

[21] Kirschmann C, Kececioglu D, Korinthenberg R, et al. Echocardiographic and electrocardiographic findings of cardiomyopathy in Duchenne and Becker-Kiener muscular dystrophies. Pediatr Cardiol 2005; 26:66–72. 10.1007/s00246-004-0689-2 15793655

[22] Vita G, DiLeo R, DeGregorio C, et al. Cardiovascular autonomic control in Becker muscular dystrophy. J Neurol Sci 2001; 186:45–49. 10.1016/s0022-510x(01)00500-7 11412871

[23] Akita H, Matsuoka S, Kuroda Y. Predictive electrocardiographic score for evaluating prognosis in patients with Duchenne’s muscular dystrophy. Tokushima J Exp Med 1993; 40:55–60. 8211981

[24] Lang, RM, Bierig M, Devereux RB, et al., Chanber Quantification Writing Group, American Society of Echocardiography’s Guidelines and Standared Committee, European Association of Echocardiography, Recommendations for chamber quantification: a report from the American Society of Echocardiography’s Guidelines and Standards Committee and the Chamber Quantification writing group, deverloped in conjuction with the European Association of Echocardiography, a branch of the European Society of Cardiology. J Am Soc Echocardiogr 2004; 18(12): 1440-63. 10.1016/j.echo.2005.10.005 16376782

[25] Nielsen JC, Lytrivi ID, Ko HH, et al.,The accuracy of echocardiographic assessment of left ventricular size in children by the 5/6 area x length (bullet) method. Echocardiogr. 2010; 27 (6); 691-5. 10.1111/j.1540-8175.2009.01120.x 20412269

