# Correction: A Randomized, Double-Blind Trial of Lisinopril and Losartan for the Treatment of Cardiomyopathy in Duchenne Muscular Dystrophy

**DOI:** 10.1371/currents.md.995441cf82fd58a29c6e4fdd5cd36b89

**Published:** 2015-03-06

**Authors:** PLOS Currents

## Correction

The fourth author was inadvertently left off the original author listing. Laurence Viollet-Callendret has been added to the original article as the fourth author.

## Reference

Allen HD, Flanigan KM, Thrush PT, Viollet-Callendret L, Dvorchik I, Yin H, Canter C, Connolly AM, Parrish M, McDonald CM, Braunlin E, Colan SD, Day J, Darras B, Mendell JR. A Randomized, Double-Blind Trial of Lisinopril and Losartan for the Treatment of Cardiomyopathy in Duchenne Muscular Dystrophy. PLOS Currents Muscular Dystrophy. 2013 Dec 12. Edition 1. doi: 10.1371/currents.md.2cc69a1dae4be7dfe2bcb420024ea865.

View Article

